# Supplementary material for: Synergistic effects of mixing and strain in high entropy spinel oxides for oxygen evolution reaction
Source: Nat Commun. 2023 Sep 23;14:5936. doi: 10.1038/s41467-023-41359-7 (PMC10517924; doi:10.1038/s41467-023-41359-7)
Supplement: Supplementary file 1 — Supplementary Information [file 41467_2023_41359_MOESM1_ESM.pdf]

Supplementary Information

Synergistic Effects of Mixing and Strain in High Entropy Spinel Oxides for Oxygen Evolution Reaction

Jihyun Baek[1,a], Md Delowar Hossain[2,3,a], Pinaki Mukherjee[4], Junghwa Lee[5,6], Kirsten T Winther[3], Juyoung Leem[1], Yue Jiang[1], William Chueh[5,6], Michal Bajdich[3,*], and Xiaolin Zheng[1,*]

[1]Department of Mechanical Engineering, Stanford University, Stanford, CA 94305, United States

[2]SUNCAT Center for Interface Science and Catalysis, Department of Chemical Engineering, Stanford University, Stanford, CA 94305, United States

[3]SUNCAT Center for Interface Science and Catalysis, SLAC National Accelerator Laboratory, Menlo Park, CA 94025, United States

[4]Stanford Nano Shared Facilities, Stanford University, CA 94305 United States

[5]Department of Materials Science and Engineering, Stanford University, Stanford, CA 94305, United States

[6]Stanford Institute for Materials and Energy Science, SLAC National Accelerator Laboratory, Menlo Park, CA, USA

[a]These authors contributed equally to this work.

Supplementary Table 1 | DFT Optimized pure bulk spinel structural and magnetic parameters.

Type	Stable spinel phase	a (Å)	b (Å)	c (Å)	Cal. vol. ($Å^3$)/ (AB_2O_4)	Exp. vol ($Å^3$)/ (AB_2O_4)	Calc. local magnetic moments (μ_B)
Cr_3O_4	Normal	5.94	5.91	6.26	77		AFM: Cr^{2+}(3.76); Cr^{3+}(3.0)
Mn_3O_4	Normal	5.59	5.90	5.90	82		AFM: Mn^{2+}(4.56); Mn^{3+}(3.86)
Fe_3O_4	Inverse	6.03	6.03	6.03	77.5	74 [1]	AFM: Fe^{3+}(4.1); {Fe^{2+}(3.74); Fe^{3+}(4.25)}
Co_3O_4	Normal	5.75	5.75	5.75	67.5	65.7 [2]	AFM: Co^{2+}(2.65); Co^{3+}(0.0)
Ni_3O_4	Normal	5.67	5.90	5.90	69.5		AFM: Ni^{2+}(1.85); Ni^{3+} (1.24)

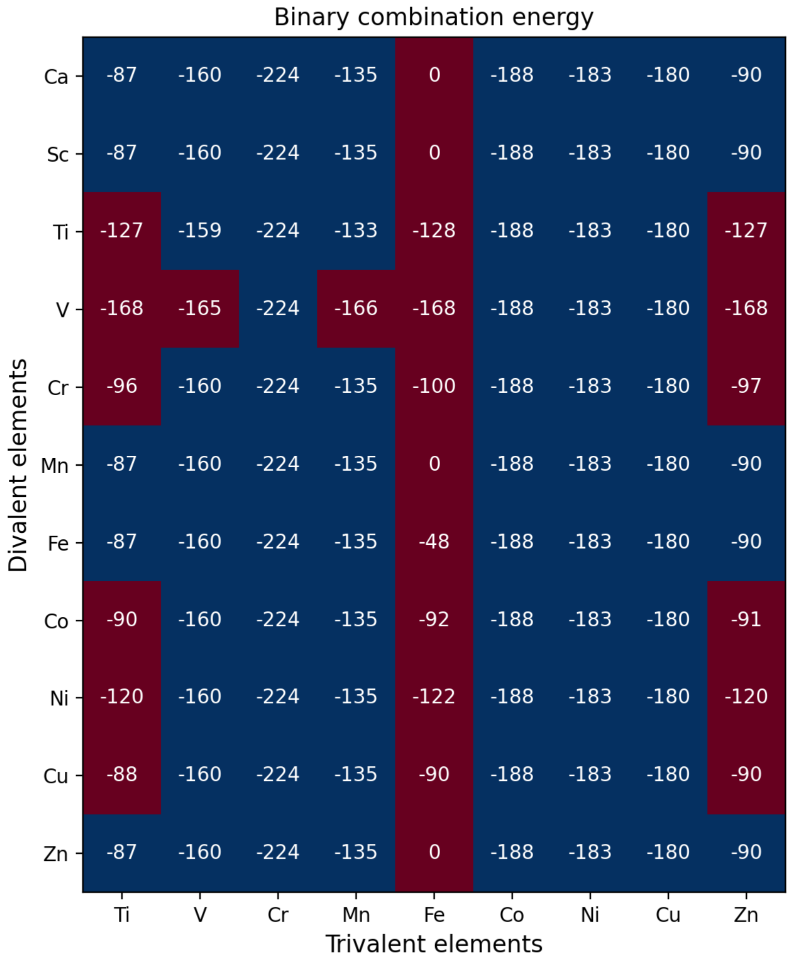

Supplementary Fig. 1 | Calculated crystal field (CF) energies for the binary combination of different elements in bulk spinel oxides. A deep blue color indicates normal (N) spinel and red color indicates inverse (I) spinel. A combination of divalent elements with trivalent Fe normally produces inverse-type spinel.

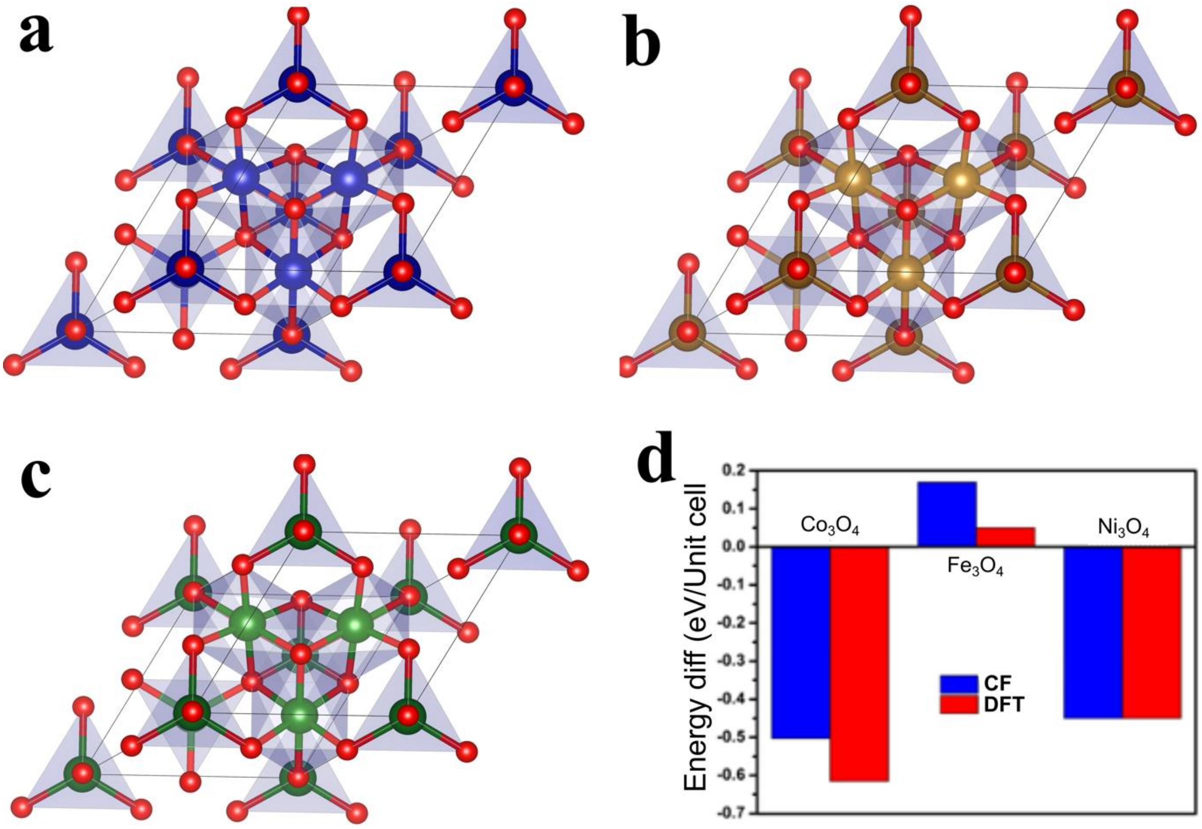

Supplementary Fig. 2 | Bulk spinel systems and their energy comparison between DFT and Crystal field (CF) theory.[3] Primitive AFM cells for **a** Normal Co_3O_4 spinel; **b** Inverse Fe_3O_4 spinel; and **c** Normal Ni_3O_4 spinel systems. **d** The energy difference between normal and inverse system comparison between DFT and CF for Co_3O_4, Fe_3O_4, and Ni_3O_4 spinel.

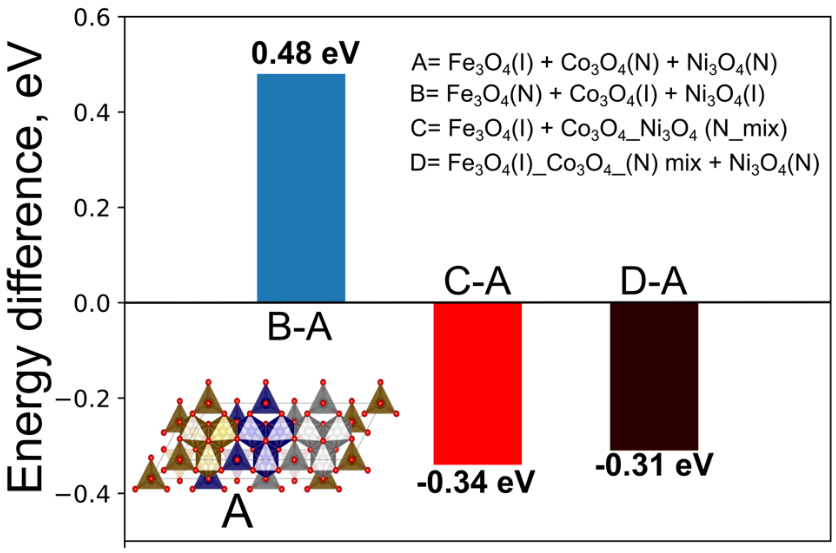

Supplementary Fig. 3 | Energy stability for the ternary elemental mixture for HEOs. We mix three (3) spinel structures and calculate binding/formation energy relative to the sum of each stable bulk structure (Fe_3O_4 Inverse, Co_3O_4 & Ni_3O_4 Normal). The B combination where we calculate individual bulk energy of Fe_3O_4 Normal, Co_3O_4 & Ni_3O_4 Inverse, and the summation energy show less stable as compared to the reference. On the other hand, C and D combinations show mixture systems where elements within the same (C) and different (D) phase swap their position. From the energy calculation, we can see that the C combination possesses the most negative energy, indicating the most stable ternary mixture.

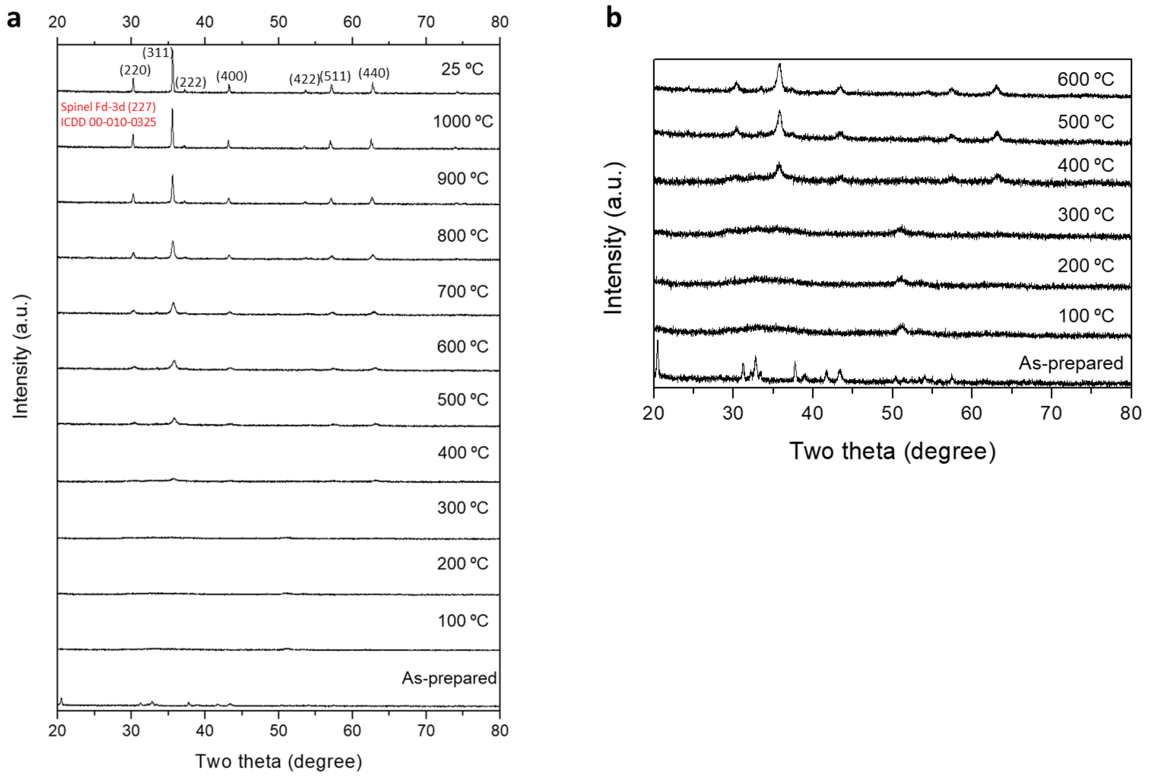

Supplementary Fig. 4 | In-situ temperature-dependent XRD for the HEO sample after combustion synthesis. a In-situ temperature-dependent XRD results with the temperature ranges from room temperature to 1000 ˚C in the air. The as-prepared result is for HEO after the combustion synthesis. The spectrum at 25 ˚C is the one cooled down after annealing at 1000 ˚C. **b** Zoom-in image ranging from room temperature to 600 ˚C. The spinel crystal structure appears after 400 ˚C.

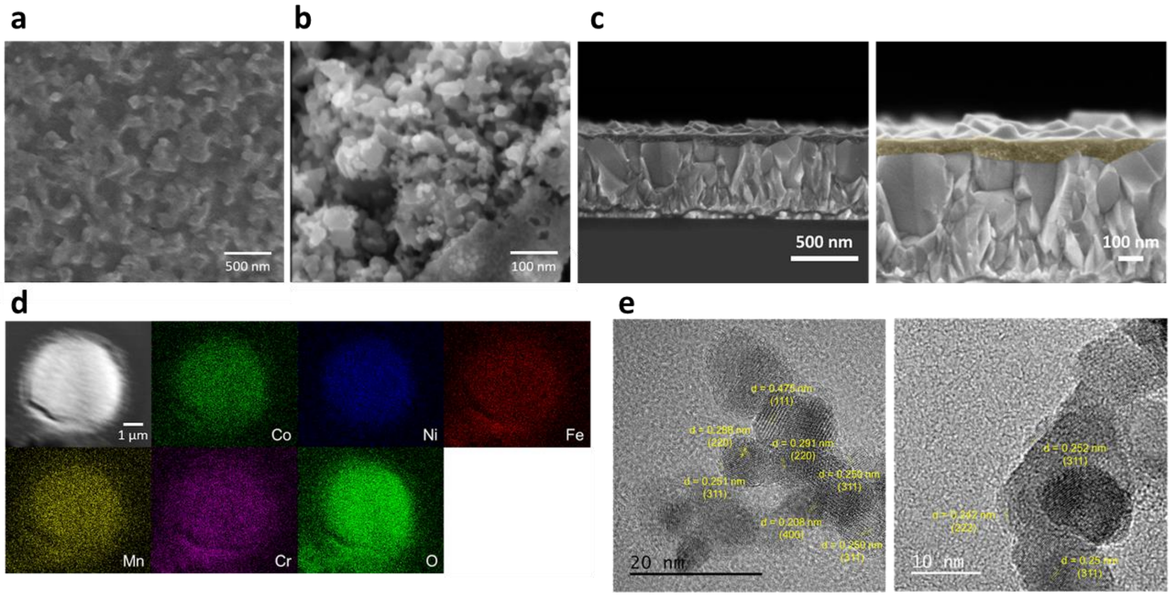

Supplementary Fig. 5 | SEM and TEM images of the HEO sample. a HEO particles on FTO/glass substrate. **b** Higher magnification SEM image of HEO particles. **c** Cross-section images of HEO film on FTO/glass substrate. **d** SEM-EDS mapping images showing all elements included. **e** Detailed TEM images.

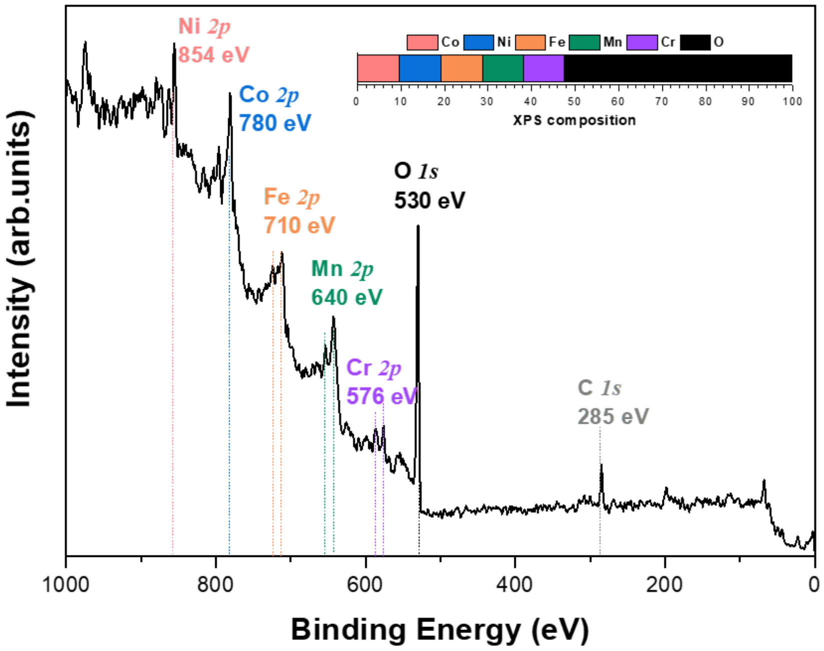

Supplementary Fig. 6 | XPS survey result of HEO particles and their elemental concentrations.

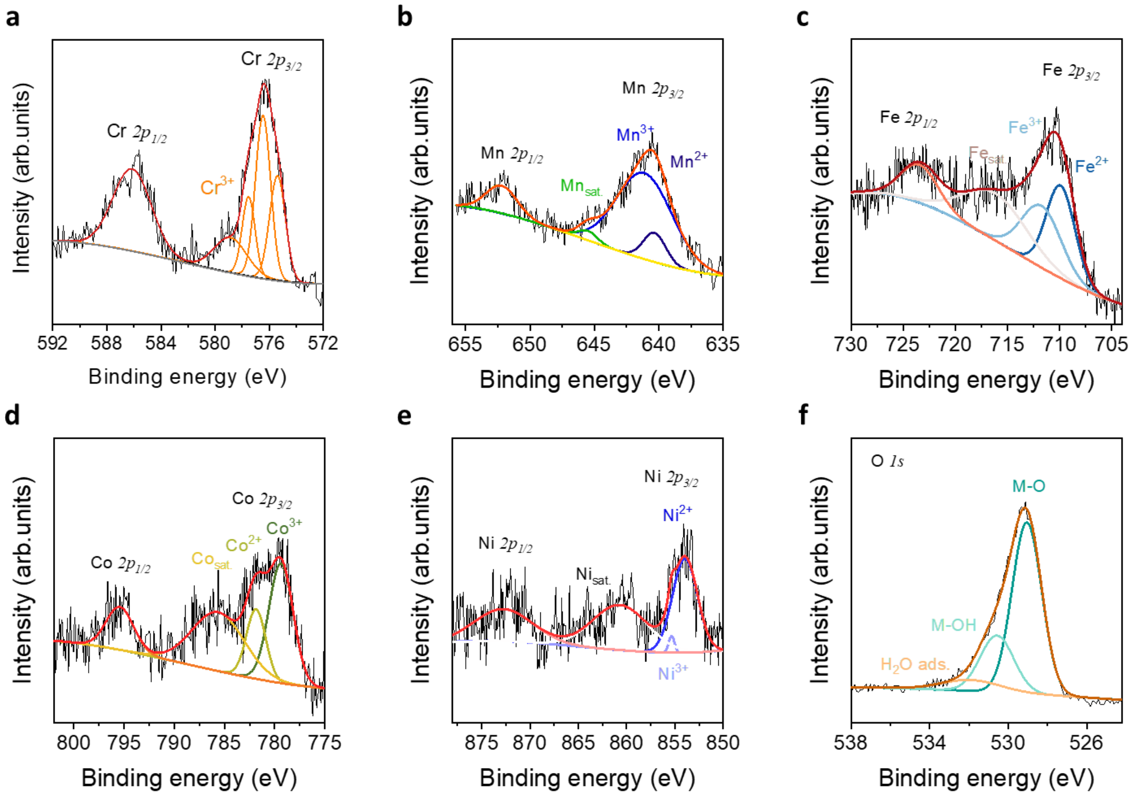

Supplementary Fig. 7 | Detailed XPS elemental survey of HEO particles. a Cr 2p, **b** Mn 2p, **c** Fe 2p, **d** Co 2p, **e** Ni 2p, and **f** O 1s. Cr has a single oxidation state (Cr^{3+}) with peaks between 575.7 and 578.9 eV in the Cr $2p_{3/2}$ region. The Mn $2p_{3/2}$ region consists of the peak at 640.1 eV corresponding to the divalent state, while the peak at 642.2 eV belongs to the trivalent state. In the Fe $2p_{3/2}$ region, the peak at 709.7 eV indicates a divalent state, while the peak at 711.2 eV shows the existence of the trivalent state. The Co $2p_{3/2}$ spectra exhibit the Co^{3+} peak at 779.6 eV and the Co^{2+} peak at 781.6 eV. Lastly, the Ni $2p_{3/2}$ has the dominant N^{2+} peak at 854.9 eV and the inferior Ni^{3+} peak at 856.5 eV. The valence state positions of all elements show a little deviation from the literature values as the HEO particle incorporates random bonding distances between the atoms.

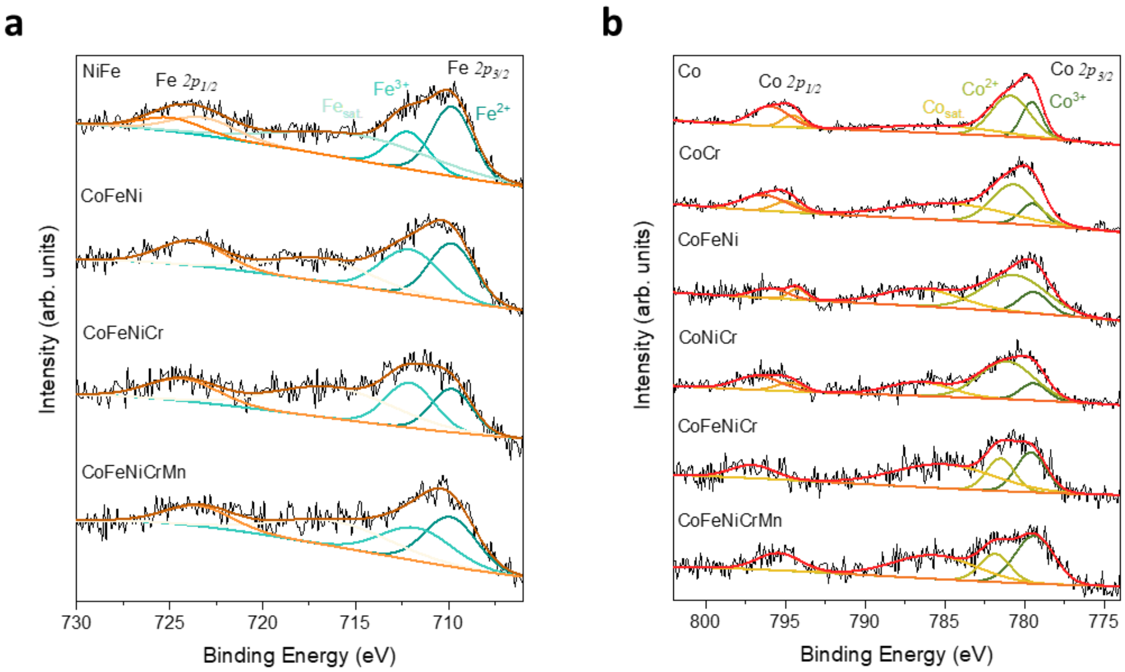

Supplementary Fig. 8 | XPS elemental survey result of Fe 2p and Co 2p for different spinel oxides. **a** Fe 2p, and **b** Co 2p to compare the amount of each oxidation state for multi-element spinel oxides.

Supplementary Table 2 | The elemental concentration of each oxidation state of each element for multi-element spinel oxides from the XPS survey analysis.

	Co^{2+}	Co^{3+}	Fe^{2+}	Fe^{3+}	Ni^{2+}	Ni^{3+}	Cr^{3+}	Cr^{6+}	Mn^{2+}	Mn^{3+}	O
Co	0.158 ± 0.032	0.202 ± 0.045									0.640 ± 0.016
Fe			0.236 ± 0.043	0.100 ± 0.011							0.663 ± 0.053
CoFe	0.058 ± 0.0003	0.071 ± 0.001	0.145 ± 0.039	0.100 ± 0.032							0.626 ± 0.008
CoFeNi	0.042 ± 0.012	0.059 ± 0.021	0.032 ± 0.013	0.079 ± 0.024	0.088 ± 0.008	0.018 ± 0.016					0.683 ± 0.051
CoFeNiMn	0.038 ± 0.003	0.060 ± 0.015	0.030 ± 0.008	0.086 ± 0.030	0.091 ± 0.020	0.008 ± 0.005			0.024 ± 0.012	0.068 ± 0.009	0.593 ± 0.051
CoFeNiCr	0.039 ± 0.009	0.065 ± 0.005	0.027 ± 0.003	0.081 ± 0.005	0.081 ± 0.006	0.018 ± 0.008	0.084 ± 0.011	0.021 ± 0.002			0.582 ± 0.026
CoFeNiCrMn	0.034 ± 0.007	0.056 ± 0.005	0.059 ± 0.016	0.035 ± 0.011	0.088 ± 0.008	0.005 ± 0.002	0.066 ± 0.011	0.022 ± 0.003	0.014 ± 0.003	0.060 ± 0.011	0.561 ± 0.025

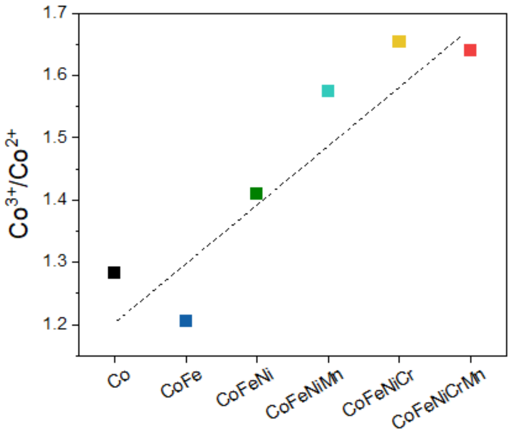

Supplementary Fig. 9 | Co^{3+}/Co^{2+} ratio based on XPS from Supplementary Table 2.

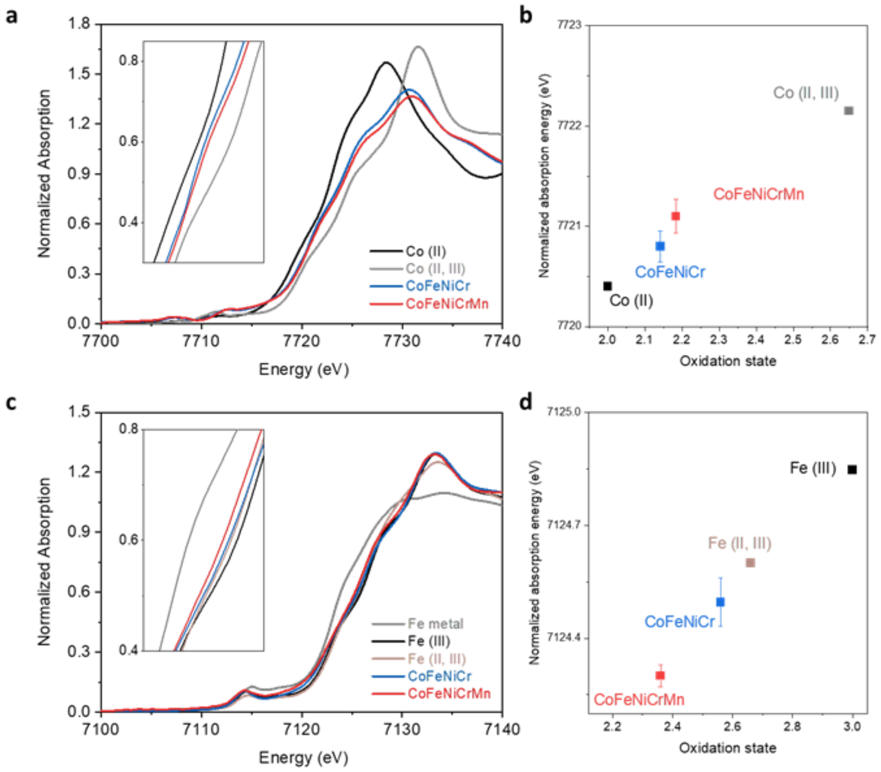

Supplementary Fig. 10 | Normalized X-ray absorption near-edge structure (XANES) analysis for HEO with other reference oxides. a XANES spectra of the Co K-edge. The inlet figure shows the expanded section from 7718 to 7725 eV. The references include CoO (Co (II)) and Co_3O_4 (Co (II, III)). **b** The calculated Co oxidation states from the XANES spectra. **c** Fe K-edge XANES spectra. The inset indicates the expanded region from 7123 to 7127 eV. The reference oxides are Fe_2O_3 (Fe (III)) and Fe_3O_4 (Fe (II, III)). **d** The calculated Fe oxidation states from the XANES spectra.Bulk transition metal Co and Fe oxidation state measured by transmission-based TM K-edge X-ray absorption spectroscopy (XAS). First, both Co and Fe oxidation states were estimated by comparing Half E_0 edge height and using the previously reported integral method, which was found to be a better descriptor of the transition metal oxidation state rather than the half-height or second derivative method. Here, the calibration curves were made using the integral method with lower and upper limits of 0.3 and 1. The slopes are the only values taken from these plots for comparing each oxidation state. The reference oxides are also listed to show the correlation between the excitation energy and metal valence.

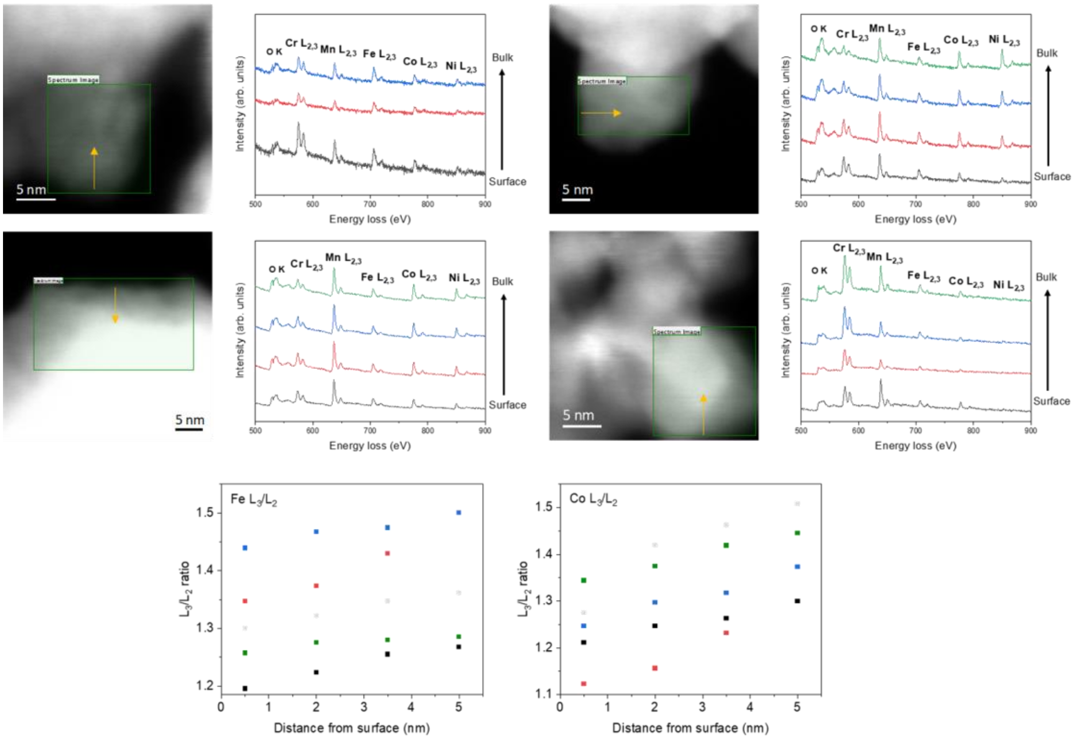

Supplementary Fig. 11 | Several spots of dark-field images of HEO particles and corresponding EEL spectra extracted from the points (3 to 4 points) with a distance of 1.5 nm between each point. The last two plots show the summarized Fe and Co L_3/L_2 ratio in which the same color of the dots is from the same particle. The average and standard deviation values are shown in **Fig. 2i**.

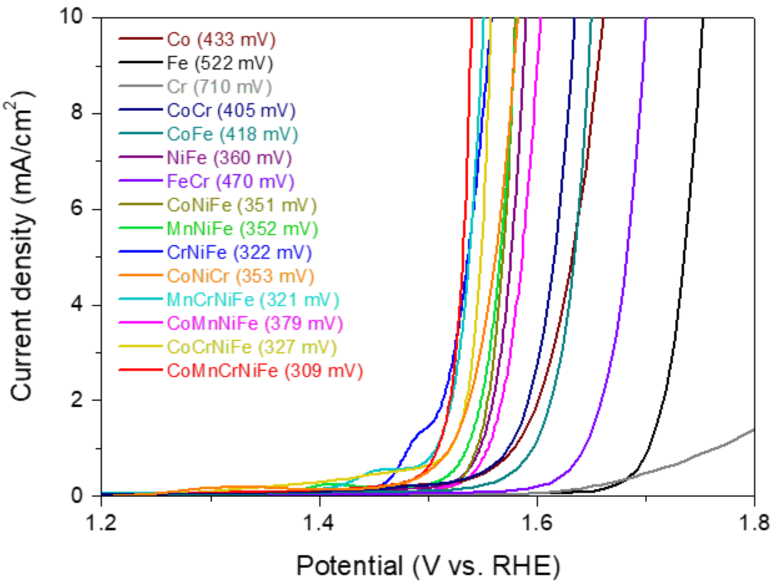

Supplementary Fig. 12 | Linear sweep voltammetry curves for all multi-element spinel oxides (from binary to senary oxides) and their overpotential value to reach 10 mA cm^{-2}.

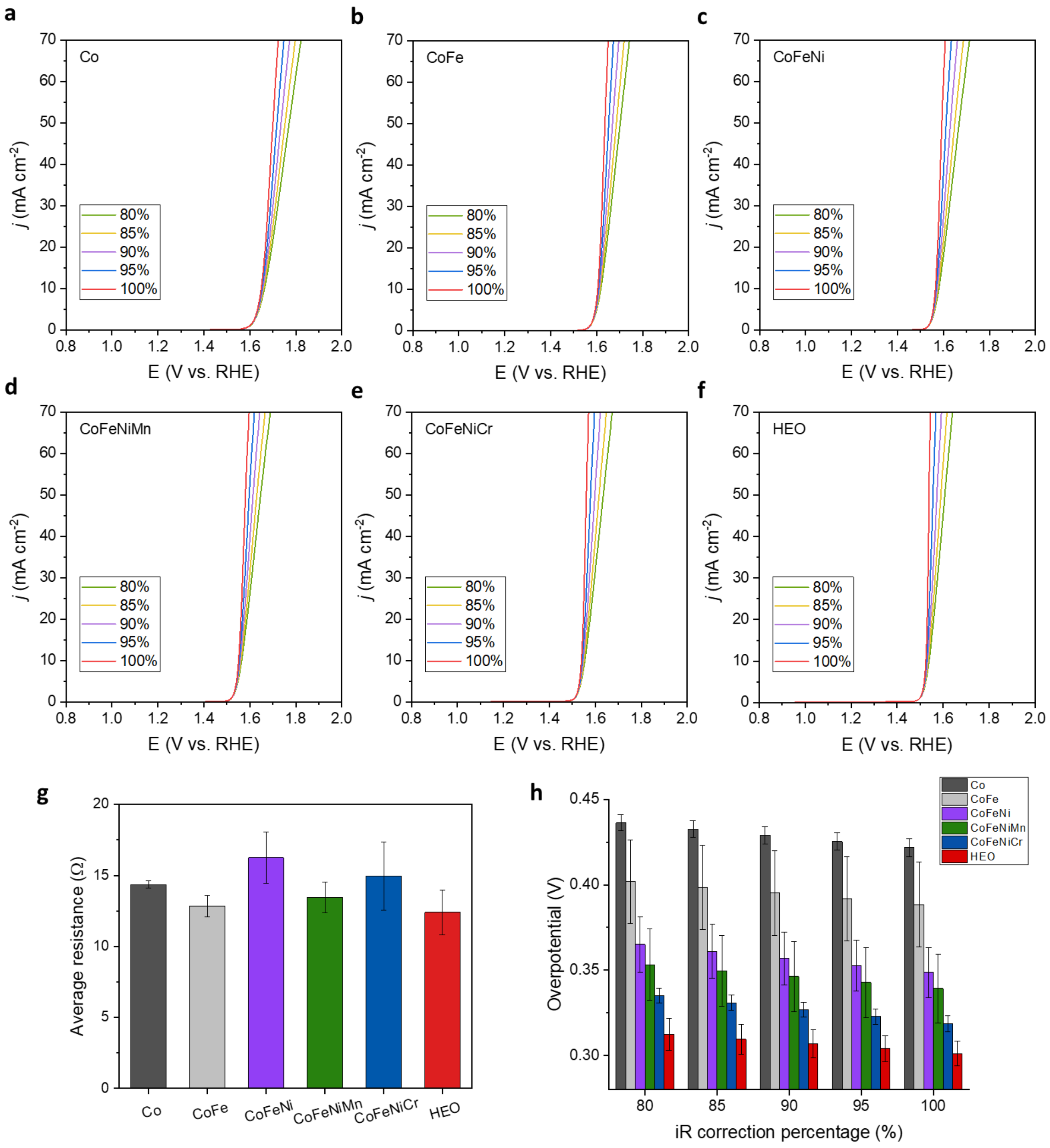

Supplementary Fig. 13 | Linear sweep voltammetry curves based on the different iR corrections. a-f LSV curves for all multi-element spinel oxides (from binary to senary oxides) with different iR corrections. **g** Average resistance values for the different spinel oxides with error bars, and **h** overpotential values with different iR correction percentages for different compositions. The error bars are evaluated from three or more samples of the same chemical composition.

Supplementary Table 3 | Overpotential value to reach 10 mA cm^{-2} in terms of different iR correction percentages.

**Sample**	**Overpotential in terms of different iR correction percentage (Error bar included in Supplementary Fig. 13)**				
	80%	85%	90%	95%	100%
Co	0.436	0.433	0.429	0.425	0.422
CoFe	0.402	0.398	0.395	0.392	0.388
CoFeNi	0.365	0.361	0.357	0.353	0.349
CoFeNiMn	0.353	0.350	0.346	0.343	0.339
CoFeNiCr	0.335	0.331	0.327	0.323	0.319
HEO	0.312	0.309	0.307	0.304	0.301

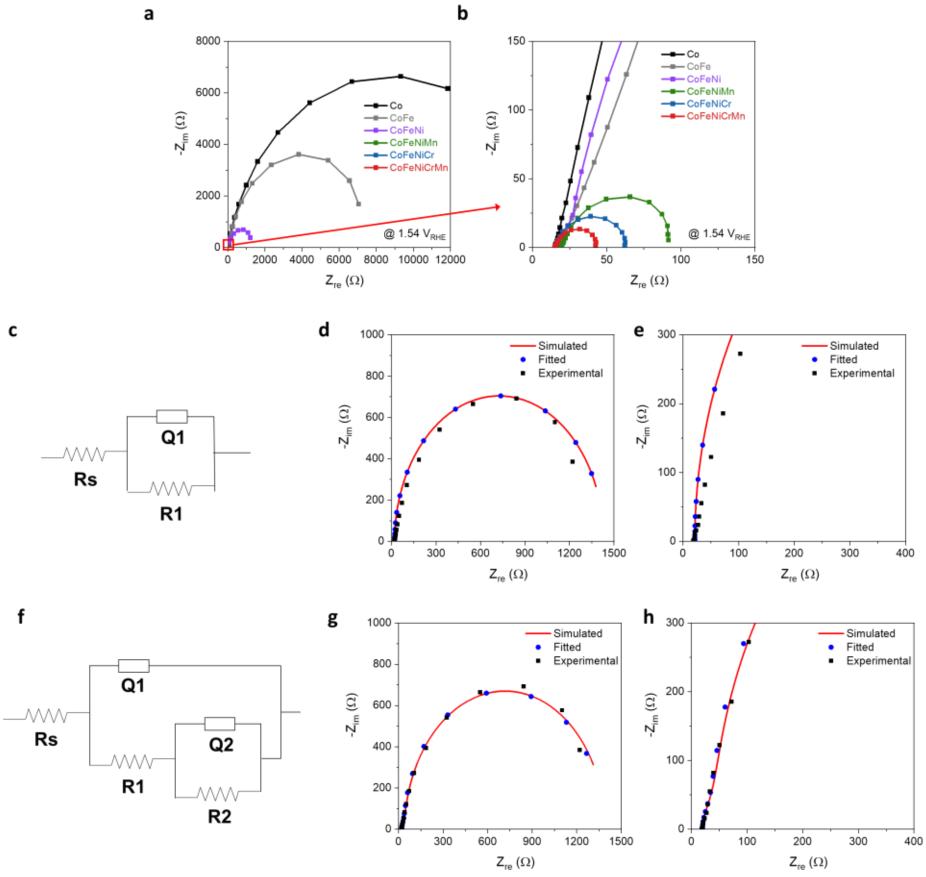

Supplementary Fig. 14 | Nyquist plots from EIS measurement for different multi-element spinel oxides and their equivalent circuit. a The EIS results for six different compositions and **b** the zoomed-in plots. The EIS results were obtained at 1.54 V vs. RHE and fitted with an equivalent circuit model in **c**. This model incorporated two types of resistances: Rs, representing any resistances associated with solution resistance and other electrical contacts, and R1, representing the charge transfer resistance at the interface between the catalyst and electrolyte. Two resistance values and double-layer capacitance are indicated in **Supplementary Table 4**. **d, e** EIS results of CoFeNi fitted by model **c**. **g, h** EIS results of CoFeNi fitted by model **f**.

Model **c** represents a simplified Randles circuit model, which characterizes a single interface between a solid and an electrolyte. On the other hand, Model **f** presents an equivalent circuit that encompasses a system with two interfaces exhibiting different kinetics. In Model **f**, additional elements (R2 and Q2) are added to the simple Randles model. This configuration is employed when the substrate electrode modified with an electrocatalyst is not fully covered, and the substrate itself also participates in the electrocatalysis process. Examples of such substrates include carbon fiber paper, glassy carbon, F-doped SnO_2, In-doped SnO_2, Ti mesh, Ni foam, and others.

Also, when Q1 > Q2, Model **f** resembles a Randles circuit and displays a single arc in the Nyquist plot, determined by the total resistance of R1+R2. If Q1 and Q2 are comparable in magnitude, the shape of the arc may appear distorted or warped due to overlapping frequency responses from each capacitor. In our specific case, although Q1 > Q2, the magnitudes are still comparable, suggesting that both models could potentially be applicable.

Supplementary Table 4 | Three resistance values for each spinel oxide from EIS analysis in Supplementary Fig. 14. This table includes resistances Rs to account for all resistances associated with solution resistance and other electrical contacts, R1 for charge transfer resistance at the interface between the catalyst and electrolyte, and Q1 for double-layer capacitance value.

	Rs (Ω)	R1 (Ω)	Q1 (F)
Co	17.10	14661	1.54e-5
CoFe	22.22	7145	1.04e-4
CoFeNi	23.06	1282	2.92e-4
CoFeNiMn	29.55	73.72	5.27e-4
CoFeNiCr	15.52	51.74	4.5e-4
CoFeNiCrMn	15.49	26.90	1.47e-3

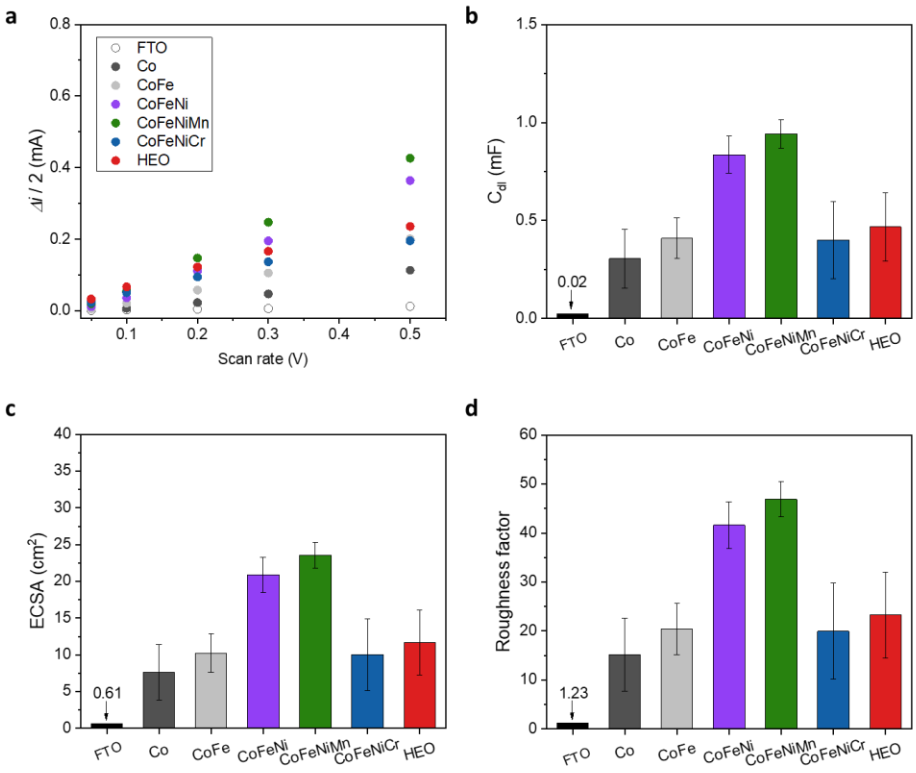

Supplementary Fig. 15 | Electrochemical surface area (ECSA) analysis. a The difference between anodic and cathodic current as a function of scan rate, and **b** the double layer capacitance values for the different spinel oxides. At least three samples were used for the measurement. **c** ECSA calculated by ECSA = C_{dl}/C_s, where C_s is the specific capacitance, which is usually used to be about 20–60 μF·cm^{-2} in alkaline solutions. We took 40 μF·cm^{-2} as the C_s value. **d** Roughness factor of the electrodes calculated by dividing ECSA by the geometric area.

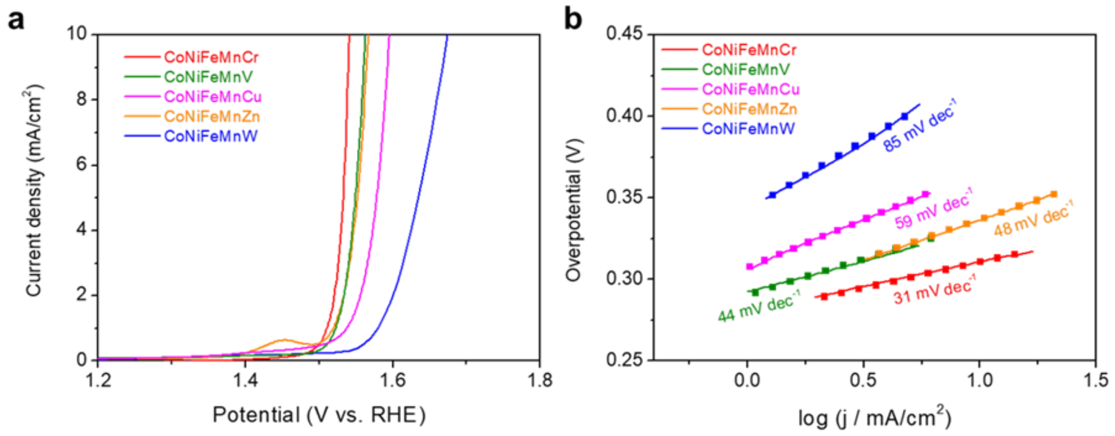

Supplementary Fig. 16 | Comparison of electrochemical activity of different senary spinel oxides. a Linear sweep voltammetry curves of five HEOs with the same CoNiFeMn but a different fifth element such as Cr, V, Cu, Zn, and W. **b** Corresponding Tafel plots of those HEOs.

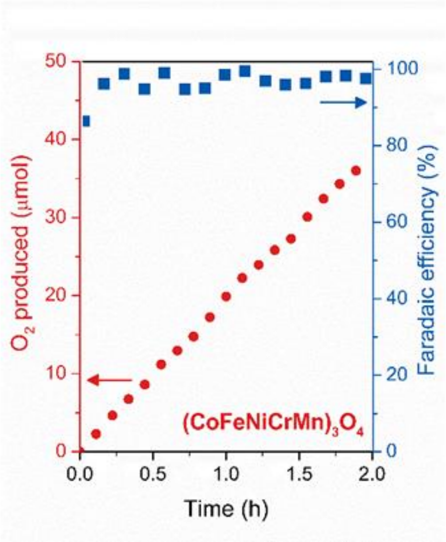

Supplementary Fig. 17 | The measured oxygen amount of the HEO sample and its Faradaic efficiency.

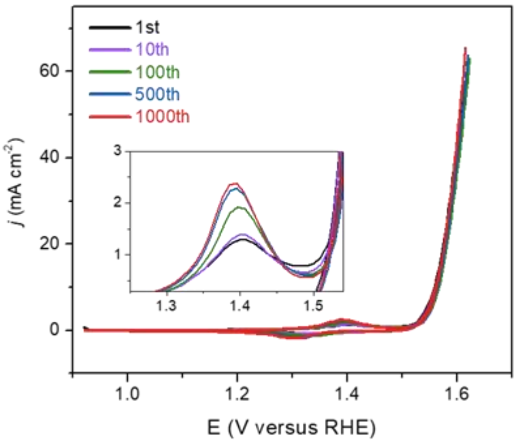

Supplementary Fig. 18 | Cyclic voltammetry of the HEO sample for 1000 cycles shows an increase in the oxidation peak around 1.4 V vs. RHE.

Supplementary Table 5 | Comparison of OER properties for various HEMs and other oxides/LDH materials.

		Catalyst	Overpotential, η (mV)	Current density	Tafel slop (mV/dec)	Stability	Electrolyte	Support material	Reference
High-entropy materials (HEMs)	1	**$(CoFeNiCrMn)_3O_4$**	**307**	**10 mA/cm^2**	**30**	**168 h**	**1 M KOH**	**FTO**	**This work**
	2	FeCoNiCrAl	317	Onset	75	-	1 M NaOH	Cu wire	4
	3	np-AlNiFeCoMo	240	10 mA/cm^2	46	50 h	1 M KOH	GCE	5
	4	$Sr_2Fe_{0.8}Co_{0.2}Mo_{0.6}Co_{0.4}O_x$	345	10 mA/cm^2	60	15 h	0.1 M KOH	Ni foam	6
	5	MnFeCoNiOx	302	10 mA/cm^2	83.7	20 h	1 M KOH	CFP	7
	6	MnFeCoNiCu-MOF	245	10 mA/cm^2	54	48 h	1 M KOH	GCE	8
	7	$(CoCuFeMnNi)_3O_4$-MWCNT	350	10 mA/cm^2	59.5	12 h	1 M KOH	GCE	9
	8	CoFeLaNiPt	377	10 mA/cm^2	150	1 h	0.1 M KOH	GCE	10
	9	AlNiCoIrMo	233	10 mA/cm^2	55.2	45 h	0.5 M H_2SO_4	GCE	11
	10	FeCoNiCrNbOx core-shell	288	10 mA/cm^2	27.7	30 h	0.1 M KOH	GCE	12
	11	$K_{0.8}Na_{0.2}(MgMnFeCoNi)F_3$	314	10 mA/cm^2	55	10 h	0.1 M KOH	GCE	13

	12	FeCoCrNi(OOH)	221	10 mA/cm^2	38.7	20 h	1 M KOH	C cloth	[14]
	13	CoCuFeMo(OOH)	199	10 mA/cm^2	48.8	72 h	1 M KOH	Cu foil	[15]
	14	$(CoCrFeMnNi)_3O_4$	288	10 mA/cm^2	60	95 h	1 M KOH	C paper	[16]
	15	$(Co_{0.2}Mn_{0.2}Ni_{0.2}Fe_{0.2}Zn_{0.2})Fe_2O_4$	326	10 mA/cm^2	53.6	10 h	1 M KOH	CFP	[17]
	16	$(CoNiMnZnFe)_3O_{3.2}$	336	10 mA/cm^2	47.5	20 h	1 M KOH	CFP	[18]
	17	$La(CrMnFeCo_2Ni)O_3$	325	10 mA/cm^2	51.2	50 h	1 M KOH	Ni foam	[19]
Binary spinel oxide	18	$Al_{0.5}Mn_{2.5}O_4$	240	25 µA/cm^2	-	-	0.1M KOH	GCE	[20]
	19	$NiCo_2O_4$	323	10 mA/cm^2	292	3 h	1 M KOH	FTO	[21]
	20	$NiFe_2O_4$	360	10 mA/cm^2	40	-	1 M KOH	C paper	[22]
	21	$Zn_{0.45}Co_{2.55}O_4$	330	10 mA/cm^2	39	10 h	1 M NaOH	Au	[23]
Binary LDH	22	Ni–Co LDH NS	670	50 mA/cm^2	113	1 h	0.1 M KOH	Ni foam	[24]
	23	Co–Ni LDH	490	1 mA/cm^2	230	400 s	0.1 M PP	FTO	[25]
	24	Co–Cr LDH	340	10 mA/cm^2	81	12 h	0.1 M KOH	GCE	[26]

	25	Zn–Co LDH	340	Onset	-	10 h	0.1 M KOH	GCE	27
	26	Co–Fe LDH	350	10 mA/cm^2	49	48 h	0.1 M KOH	GCE	28
	27	Co-Mn LDH	325	10 mA/cm^2	43	14 h	1 M KOH	GCE	29

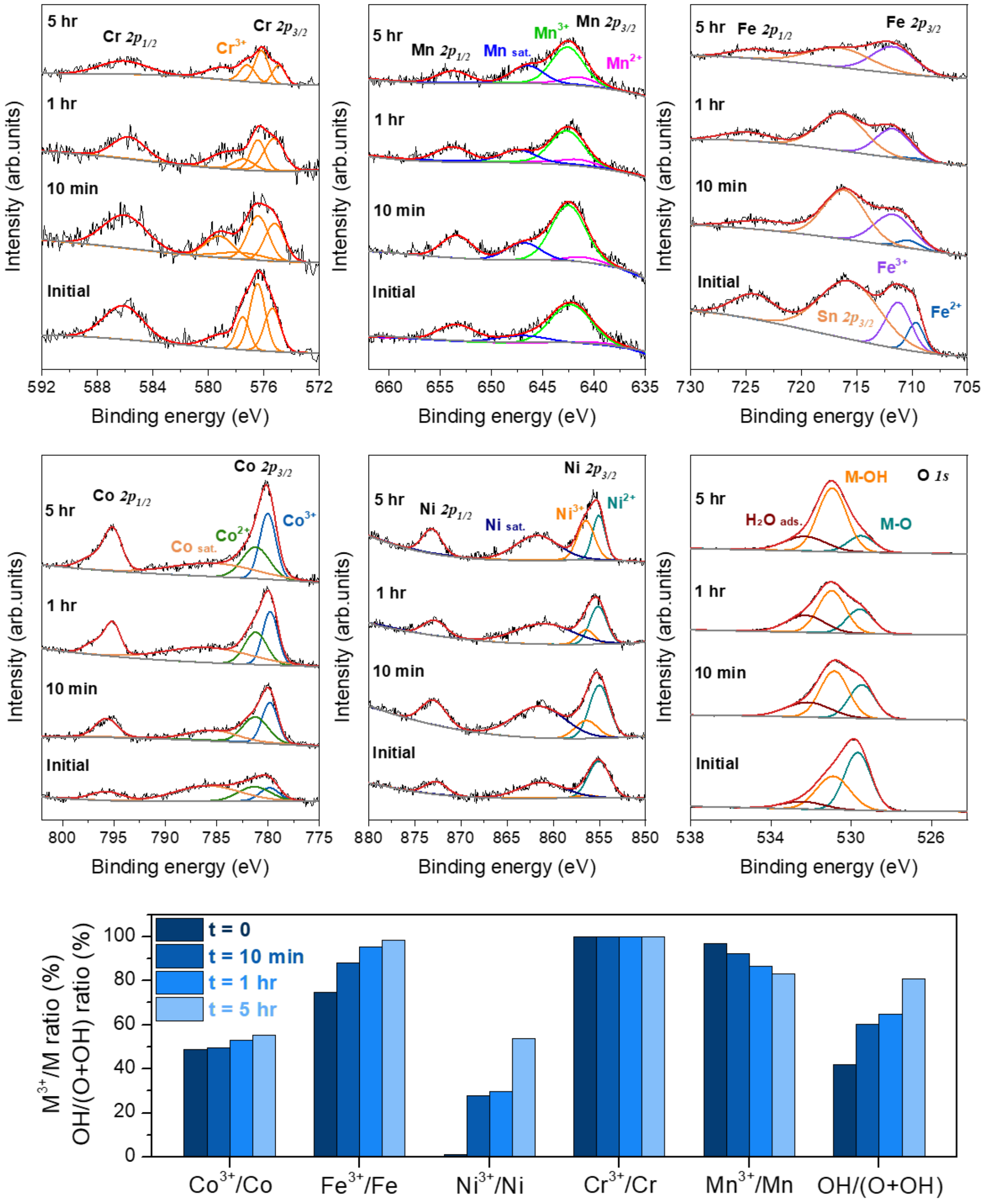

Supplementary Fig. 19 | Ex-situ XPS elemental surveys show that the oxidation state of the HEO sample (CoFeNiCrMn) changes over time with more trivalent states and OH groups.

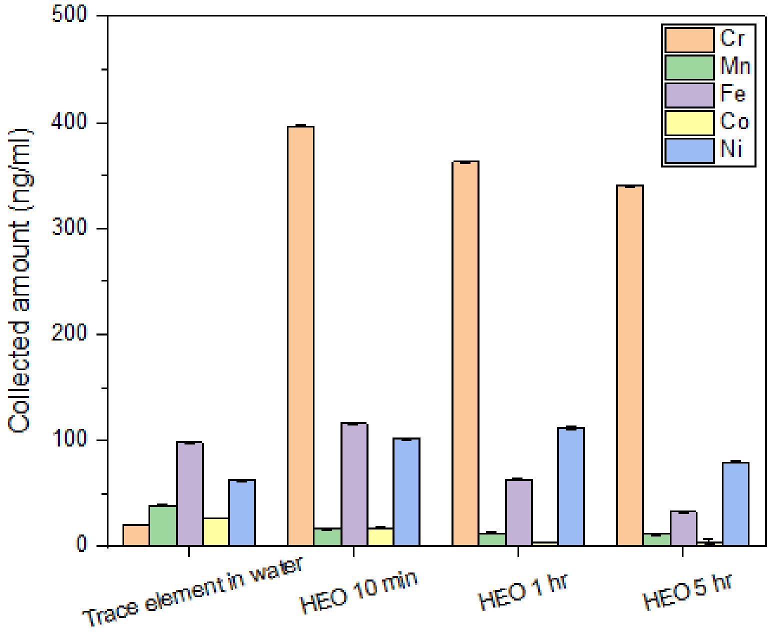

Supplementary Fig. 20 | Inductively coupled plasma mass spectrometry (ICP-MS) analysis. ICP-MS results for trace elements in the water reported by the National Institute of Standards & Technology (NIST) and the dissolved elements in the 1 M KOH electrolyte after the electrochemical measurements (10 min, 1 hour, and 5 hours).

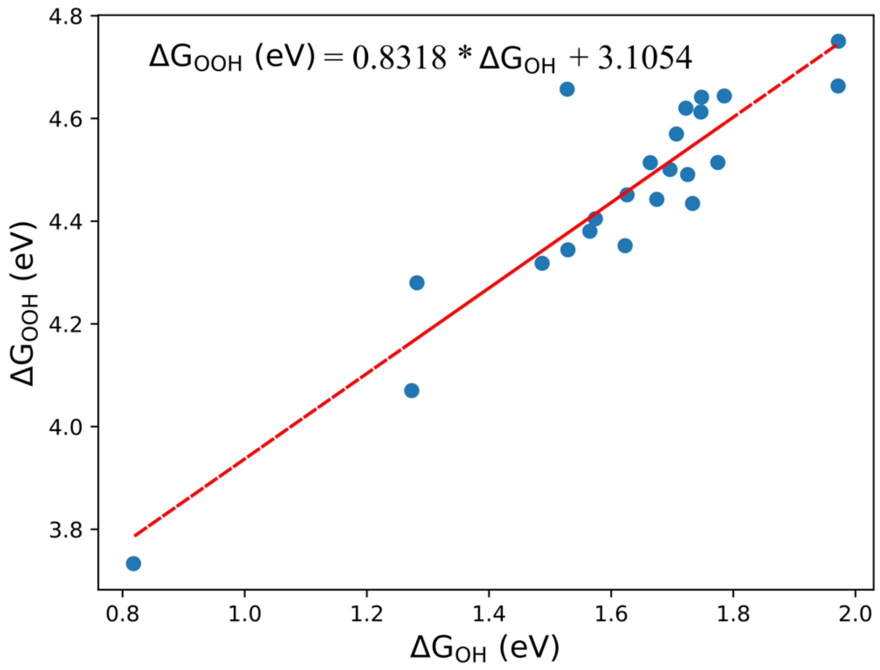

Supplementary Fig. 21 | Scaling relationship for *OOH and *OH. For OOH*, we randomly chose a few pure and HEO samples.

Supplementary Table 6 | OH*, O* and *OOH binding energies for pure spinel (100) surfaces at octahedral site. DDE is defined as the difference between DG and DE calculated relative to H_2O(l) and H_2(g) at 300K. The calculated DEs of all HEO intermediates (+1000 rows) are listed at https://www.catalysis-hub.org/publications/HossainInvestigation2022.

Pure spinel surface (Octahedral site)	ΔG_{OH*} (eV) ΔΔE=0.339	ΔG_{O*} (eV) ΔΔE=0.001	ΔG_{OOH*} (eV) ΔΔE=0.364	Overpotential (V)
Cr_3O_4	1.0571	2.1923	4.0502	0.63
Mn_3O_4	1.5302	3.6729	4.6622	0.91
Fe_3O_4	0.8509	3.491	6.4691	1.76
Co_3O_4	1.3459	3.2195	4.1907	0.64
Ni_3O_4	2.0582	4.5396	4.8576	1.25

OER mechanism in acidic conditions:[30]

Overall reaction: $2H_2O(l) \rightarrow O_2 + 4H^+ + 4e^-;\ \Delta G_0 = 4.92\ eV$ (1)

Where (*l*) represents a liquid medium. It involves four steps as follows,

$$H_2O(l) \rightarrow\ HO^* + H^+ + e^-, \quad (2)$$

$$HO^* \rightarrow O^* + H^+ + e^-, \quad (3)$$

$$H_2O(l) +\ O^* \rightarrow\ HOO^* + H^+ + e^-, \quad (4)$$

$$HOO^* \rightarrow * + O_2(g) + H^+ + e^-, \quad (5)$$

Where, * and (*g*) represent the active surface and gas phase, respectively.

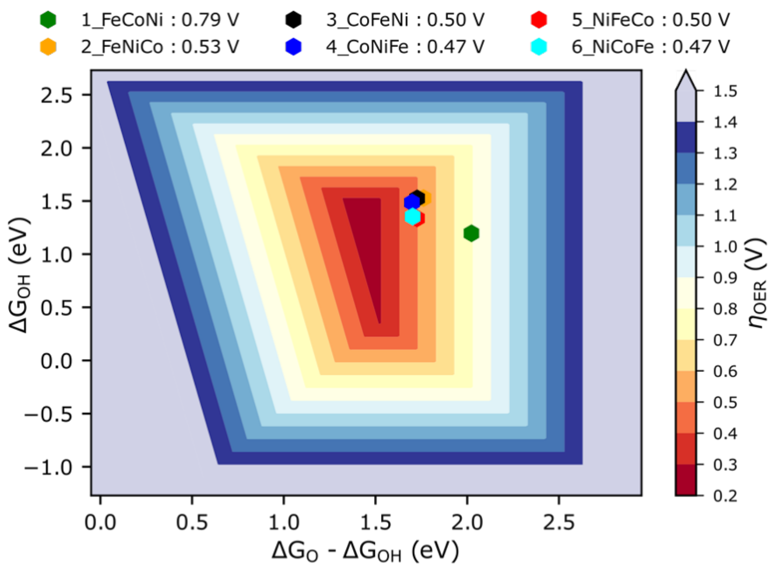

Supplementary Fig. 22 | OER activity volcano plot as a 2D heat map of overpotentials for CoFeNi oxide system based on binding energy calculation of O* and OH* and scaled OOH* values for HEO systems. We considered 3*3 permutation ($_3P_3$) for Co active sites and found 6 different combinations. Among 6 combinations, we found the lowest overpotential of 0.47 V for the Co active site.

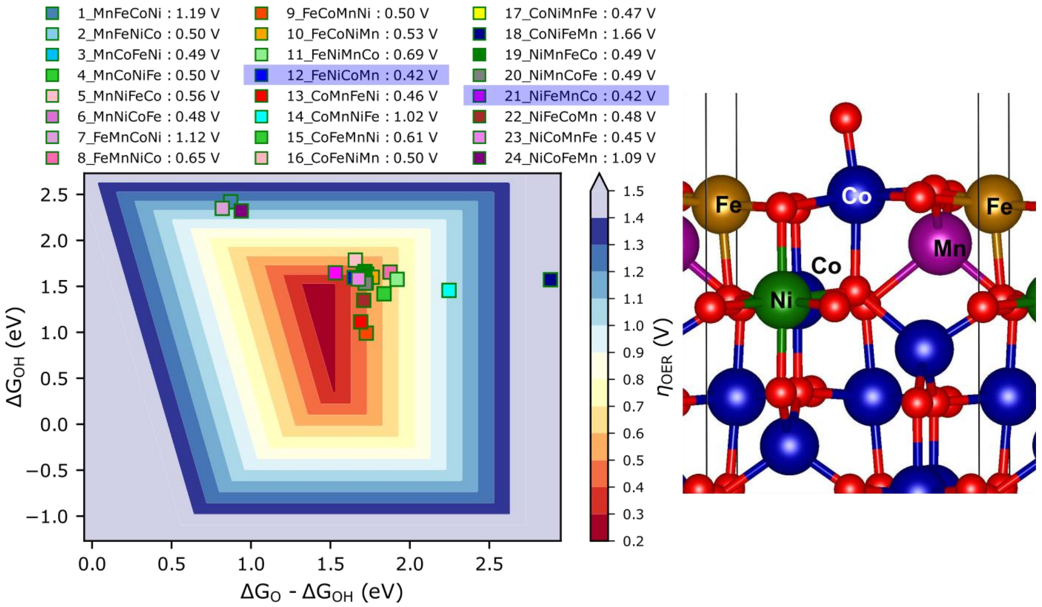

Supplementary Fig. 23 | OER activity volcano plot as a 2D heat map of overpotentials for CoFeNiMn oxide system based on binding energy calculation of O* and OH* and scaled OOH* values for HEO systems. We considered 4*4 permutation ($_4P_4$) for Co active sites and found 24 different combinations. Among 24 combinations, we predicted the lowest overpotential of 0.42 V for the Co active site.

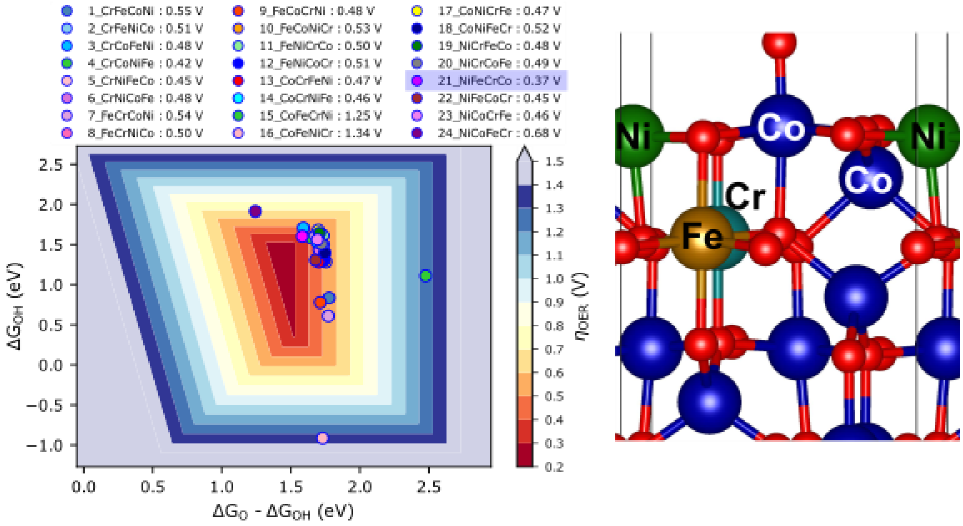

Supplementary Fig. 24 | OER activity volcano plot as a 2D heat map of overpotentials for CoFeNiCr oxide system based on binding energy calculation of O* and OH* and scaled OOH* values for HEO systems. We considered 4*4 permutation ($_4P_4$) for Co active sites and found 24 different combinations. Among 24 combinations, we predicted the lowest overpotential of 0.37 V for the Co active site.

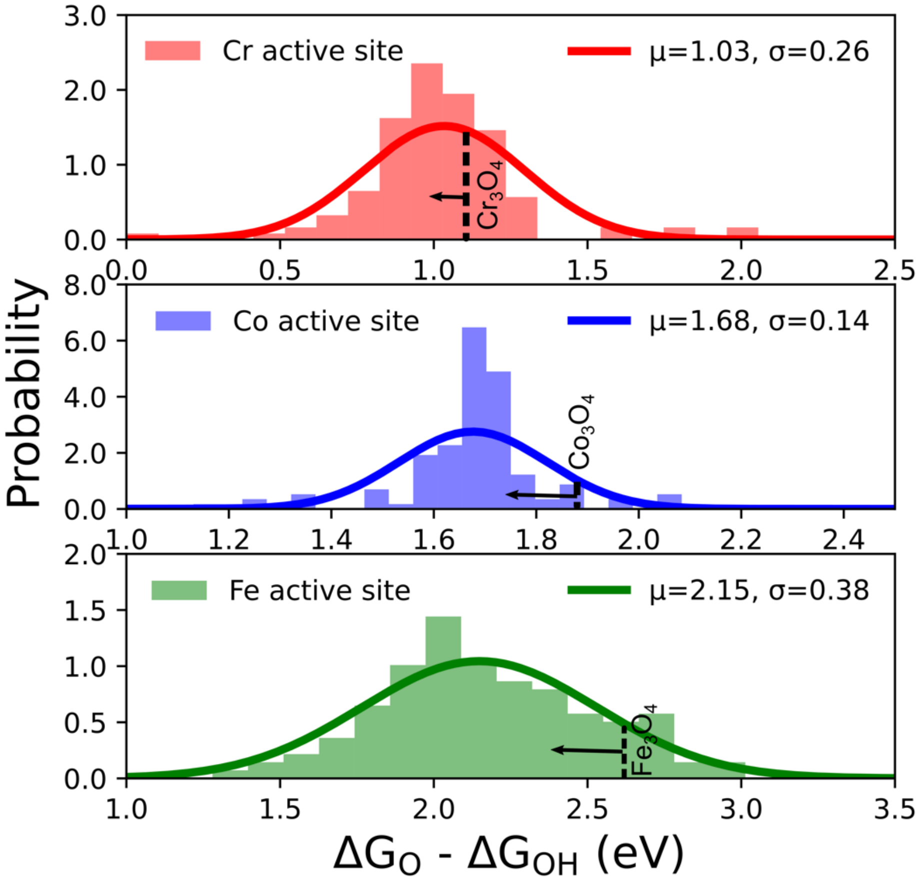

Supplementary Fig. 25 | The O*-OH* binding energy distribution for Cr, Co, and Fe active sites in the HEO system. It shows that the O*-OH* binding energy decreased (shifted) in the presence of surrounding neighboring elements in the HEO system compared to their pure spinel system. As a result, the OER minimum overpotential decreases for the Co site to 0.29 V from 0.64 V (pure), the Cr site to 0.34 V from 0.63 V (pure), and the Fe site to 0.34 V from 1.76 V (pure).

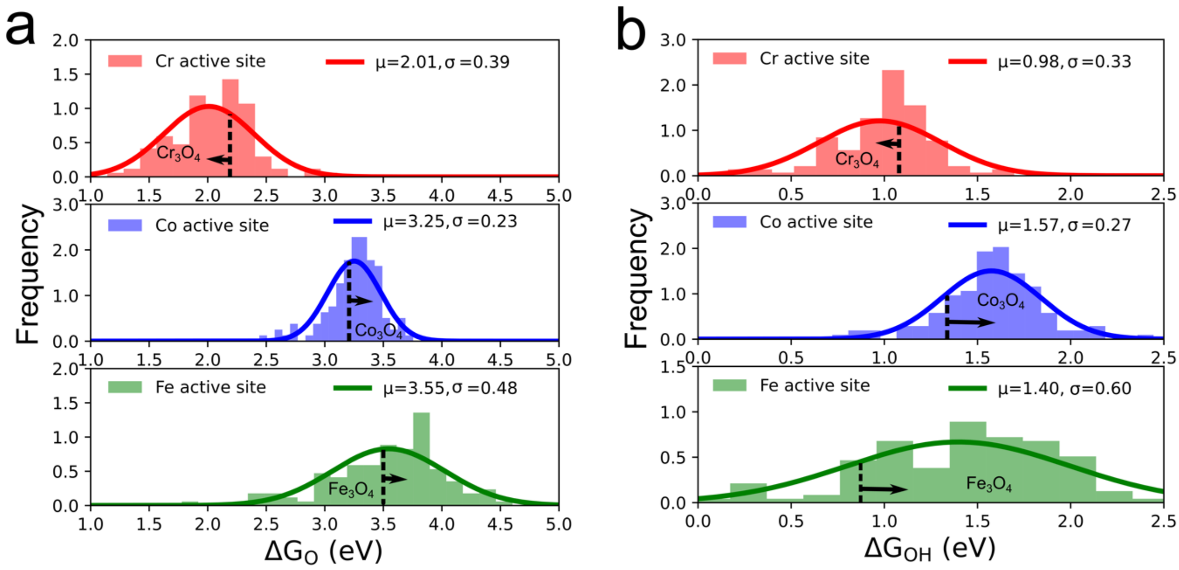

Supplementary Fig. 26 | The O* and OH* binding energy distribution for Cr, Co, and Fe active sites in the HEO system. a, b The shifts are left direction for Cr indicating bonding getting stronger while Co and Fe side shifts are right side direction indicating weak adsorption. We have conducted a total number of 120×3×3 = 1080 calculations, including clean surface, O*, and OH* systems for three active sites. The free energy of O* adsorption varies from 0.5 to 3.0 eV, 2.4 to 3.8 eV, and 2.4 to 4.6 eV, respectively, for the Cr, Co, and Fe active sites in our HEO models. The mean of free energy of ads. for Cr site (2.01 eV) is more slightly shifted to the left than for pure Cr_3O_4 pure system (2.19 eV), indicating much stronger binding to O*. The average binding of O* over the Co and Fe sites (3.25 eV and 3.55 eV) is weaker than their pure system (Co_3O_4: 3.22 eV, Fe_3O_4: 3.49 eV). On the other hand, the OH* adsorption free energy varies from 0.3-1.7 eV with an average mean of 0.98 eV for the Cr active site in HEO, shifted towards the left from 1.06 eV for the pure system. Oppositely, OH* free energies vary between 0.9-2.2 eV with a mean of 1.57 eV for Co sites and 0.2-2.5 eV with a mean of 1.40 eV for Fe sites, respectively. In both cases, OH* binding energies moved to the right side, indicating weaker interaction than pure Co_3O_4 (1.35 eV) and Fe_3O_4 (0.85 eV) systems. The large shift of >0.5 eV in the case of the Fe site might be due to the consideration of the normal octahedral site as an active site for HEO as compared to the inverse octahedral site in pure spinel. However, the shift of binding energy is not limited to one direction, which results in wide Gaussian distribution.

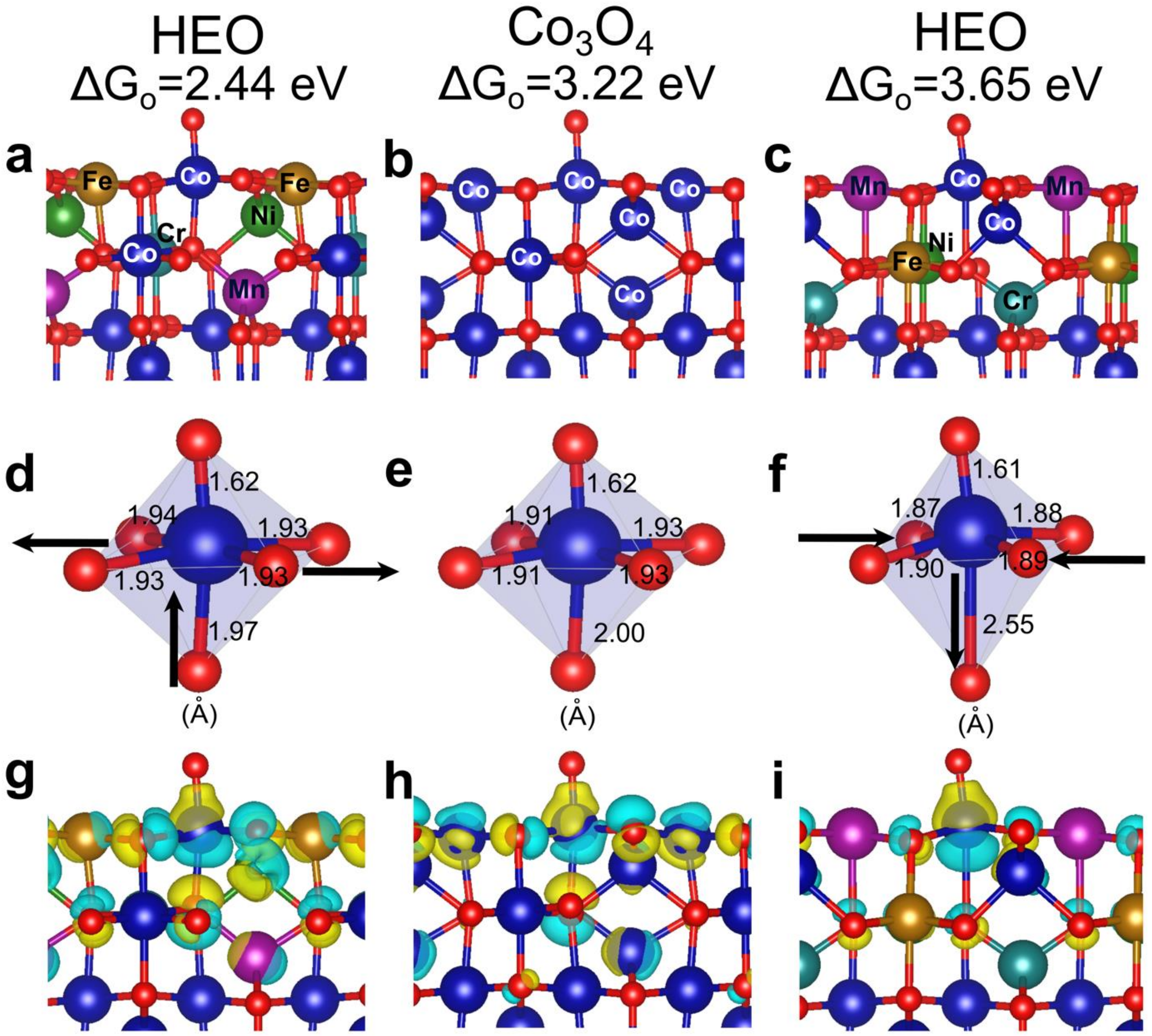

Supplementary Fig. 27 | The O* binding energy and charge density plot for Co active site in HEO and pure system. The HEO system with strong O* binding energy in **a** pure spinel Co_3O_4 surface in **b** HEO system with weak O* binding energy. The strong binding resulted from the lateral expansion of Co-O bonds in **d** than the pure system in **e**; while weak binding resulted from lateral expansion in **f** of Co-O bonds. The charge density plots show that the local environment of active sites is mostly electron-deficient, resulting in strong O* binding in **g** than pure in **h** and a weak O* binding system in **i**. (iso-surface value 0.04 e/Å^3. Cyan: charge depletion and yellow: charge accumulation)

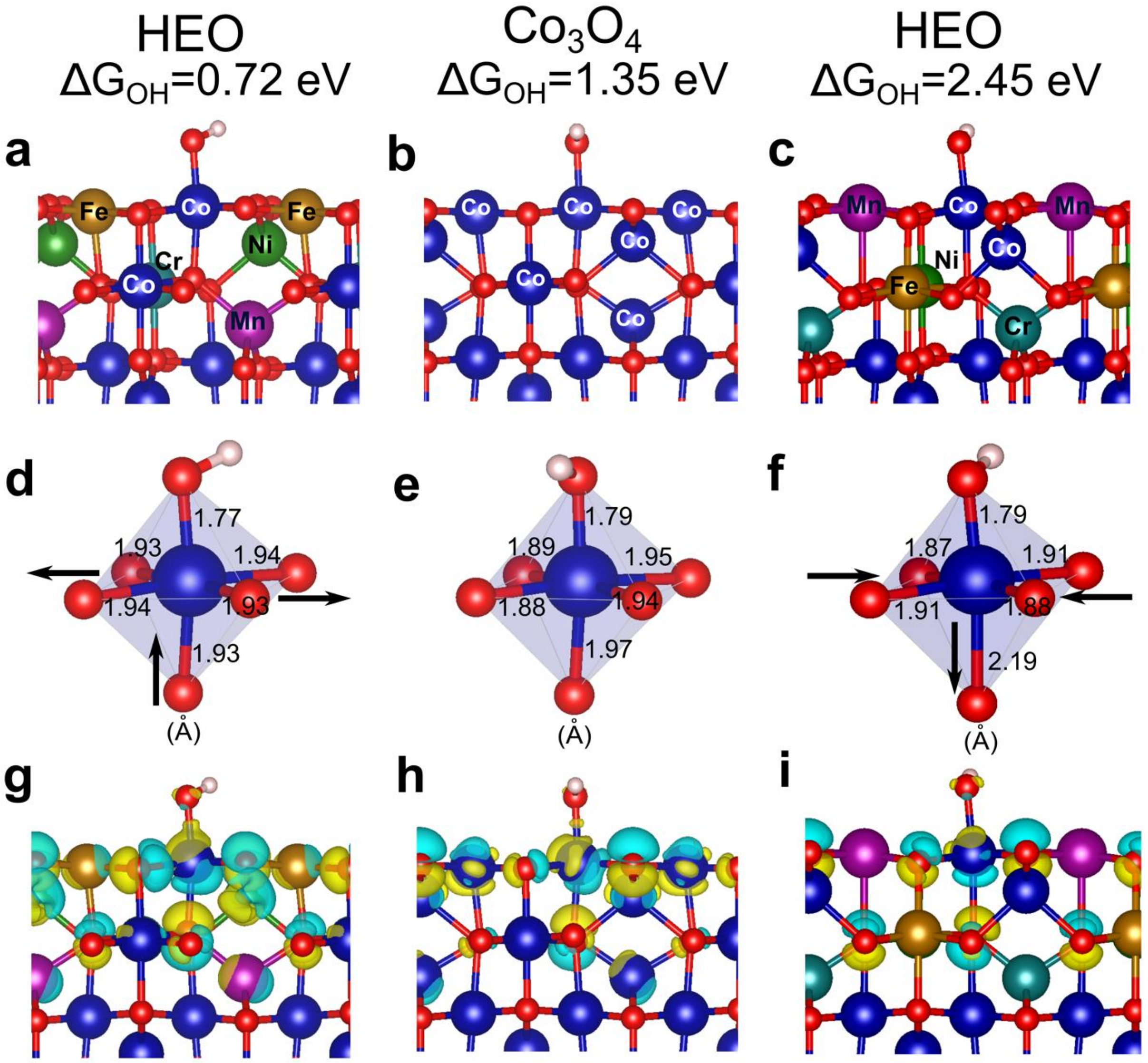

Supplementary Fig. 28 | The OH* binding energy and charge density plot for Co active site in HEO and pure system. The HEO system with strong OH* binding energy in **a** pure spinel Co_3O_4 surface in **b** HEO system with weak OH* binding energy in **c**. The strong binding resulted from the lateral expansion of Co-O bonds in **d** than the pure system in **e** while weak binding resulted from lateral expansion in **f** of Co-O bonds. The charge density plots show that the local environment of active sites is mostly electron-deficient, resulting in strong OH* binding in **g** than pure in **h** and a weak OH* binding system in **i**. (iso-surface value 0.04 e/Å^3. Cyan: charge depletion and yellow: charge accumulation)

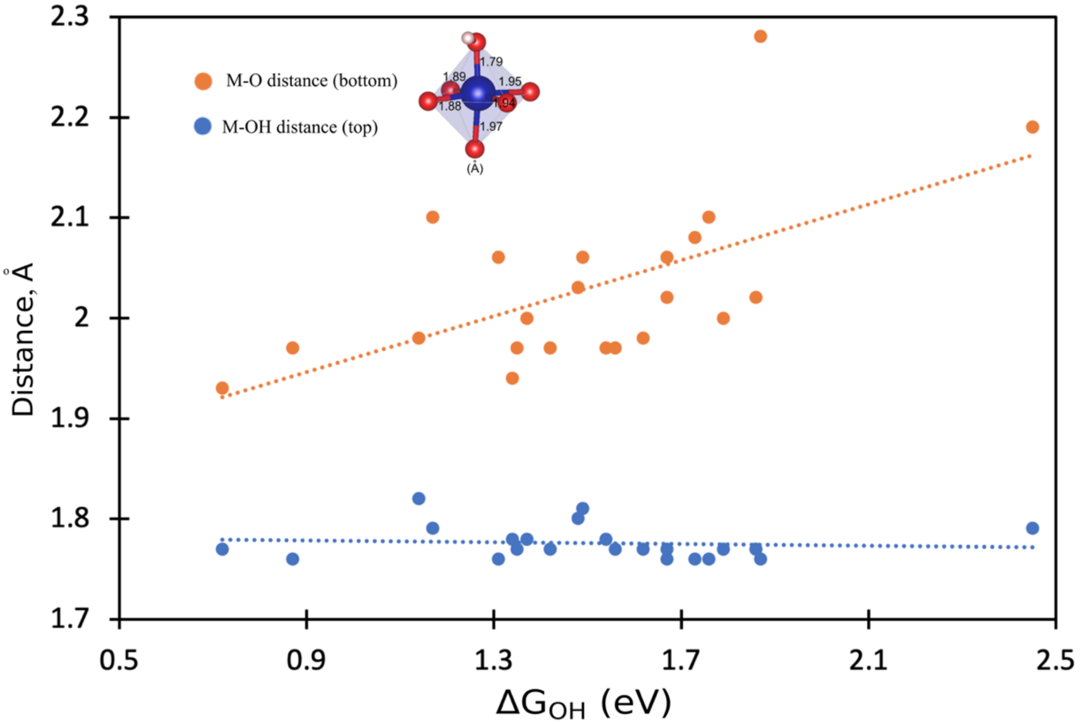

Supplementary Fig. 29 | OH-M-O distance relationship as a function of *OH binding energy. The figure shows that the top *OH bond remains almost constant despite the binding energy changes, while the lower oxygen-metal bonds increase with increasing binding energies.

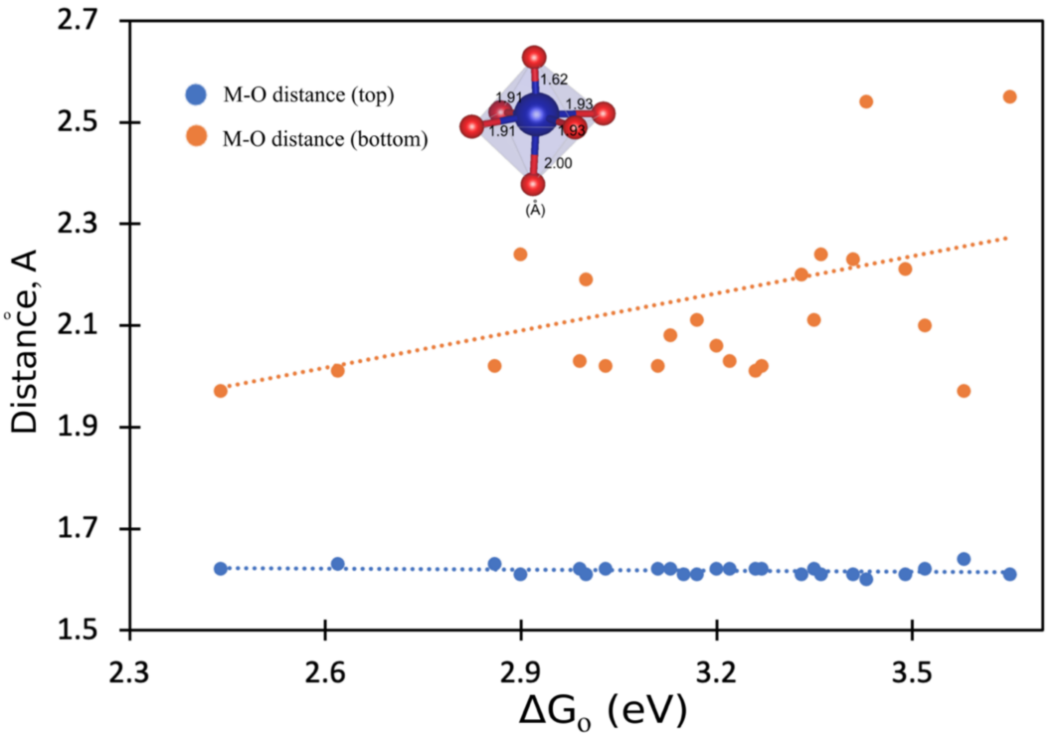

Supplementary Fig. 30 | O-M-O distance relationship as a function of *O binding energy. The figure shows that the lower M-O bond linearly increases with increasing *O binding energies while top M-O bonds remain constant across the *O binding energies.

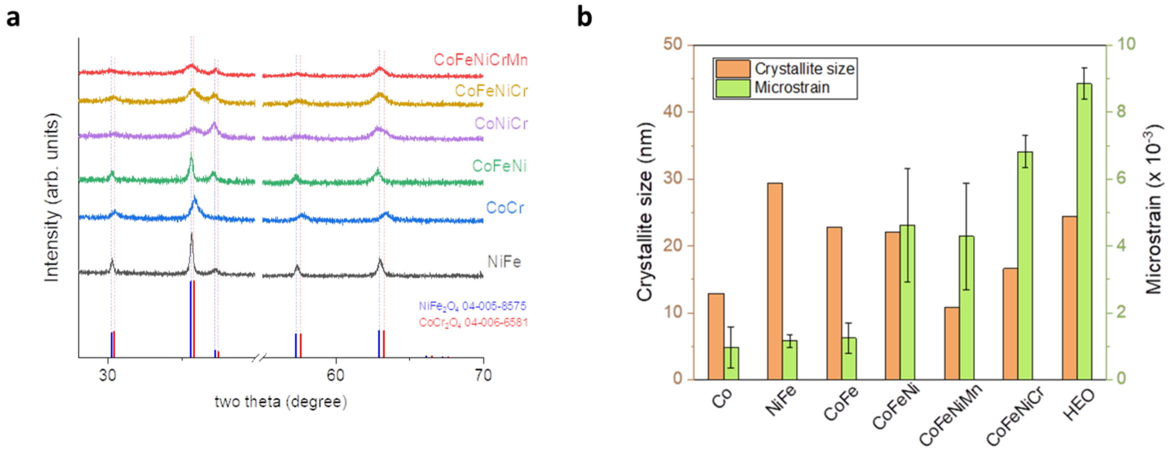

Supplementary Fig. 31 | Estimation of microstrain and crystallite size from XRD. a XRD result for several spinel oxides and **b** calculated crystallite size and microstrain for each oxide. Here, we used the Scherrer equation and Williamson-Hall plot to estimate the grain size and microstrain,

$$D = \frac{K\lambda}{\beta cos\Theta}$$

, where D is grain size, K = 0.9 (Scherrer constant), λ = 0.15406 nm, the wavelength of the x-ray sources (Cu Kα), β = FWHM (in radians), and Θ = peak position (in radians).

In the Williamson-Hall plot,

$$\beta\ cos\Theta\ =\ \varepsilon(4sin\Theta)\ +\ \frac{K\lambda}{D}$$

, where ε = microstrain. Finally, by plotting $\beta\ cos\Theta$ on the y-axis against $4sin\Theta$ on the x-axis, we can get the strain component from the slope and the particle size component from the y-intercept.

Supplementary Table 7 | Crystallite size and microstrain for different spinel oxides calculated from XRD results in Supplementary Fig. 31.

	Crystallite size (nm)	Microstrain
Co	12.91	0.97
CoFe	22.81	1.25
CoFeNi	22.04	4.62
CoFeNiMn	10.79	4.29
CoFeNiCr	16.61	6.83
HEO	24.50	8.85

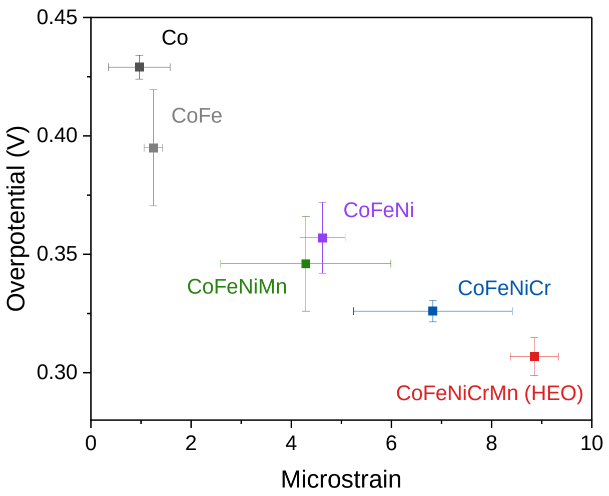

Supplementary Fig. 32 | Correlation plot of OER activity-microstrain. The x-axis represents the microstrain with the horizontal error bars and the y-axis means the overpotential values with the vertical error bars

References

1. Fleet, M. E. The structure of magnetite. *Acta Cryst.* 1981, B**37**, 917-920
2. Kotousova, I. S. & Polyakov, S. M. Electron-diffraction study of Co_3O_4 (1972)
3. Burns, R. G. Mineralogical applications of crystal field theory. Second edition. *Mineralogical applications of crystal field theory. 2nd edition* (1993)
4. Cui, X., Zhang, B., Zeng, C. & Guo, S. Electrocatalytic activity of high-entropy alloys toward oxygen evolution reaction. *MRS Commun.* 2018, **8**, 1230-1235
5. Qiu, H. J. *et al.* Noble Metal-Free Nanoporous High-Entropy Alloys as Highly Efficient Electrocatalysts for Oxygen Evolution Reaction. *ACS Materials Lett.* 2019, **1**, 526-533
6. Sun, H. *et al.* Smart Control of Composition for Double Perovskite Electrocatalysts toward Enhanced Oxygen Evolution Reaction. *ChemSusChem* 2019, **12**, 5111-5116
7. Dai, W., Lu, T. & Pan, Y. Novel and promising electrocatalyst for oxygen evolution reaction based on MnFeCoNi high entropy alloy. *J. Power Sources* 2019, **430**, 104-111
8. Zhao, X. *et al.* Ambient fast, large-scale synthesis of entropy-stabilized metal-organic framework nanosheets for electrocatalytic oxygen evolution. *J. Mater. Chem. A* 2019, **7**, 26238
9. Wang, D. *et al.* Low-temperature synthesis of small-sized high-entropy oxides for water oxidation. *J. Mater. Chem. A* 2019, **7**, 24211
10. Glasscott, M. W. *et al.* Electrosynthesis of high-entropy metallic glass nanoparticles for designer, multi-functional electrocatalysis. *Nat. Commun.* 2019, **10**, 2650
11. Jin, Z. *et al.* Nanoporous Al-Ni-Co-Ir-Mo High-Entropy Alloy for Record-High Water Splitting Activity in Acidic Environments. *Small* 2019, **15**, 1904180
12. Ding, Z. *et al.* High Entropy Intermetallic–Oxide Core–Shell Nanostructure as Superb Oxygen Evolution Reaction Catalyst. *Adv. Sustain. Syst.* 2020, **4**, 1900105
13. Wang, T., Chen, H., Yang, Z., Liang, J. & Dai, S. High-Entropy Perovskite Fluorides: A New Platform for Oxygen Evolution Catalysis. *J. Am. Chem. Soc.* 2020, **142**, 4550-4554
14. Zhang, N. *et al.* Lattice oxygen activation enabled by high-valence metal sites for enhanced water oxidation. *Nat. Commun.* 2020, **11**, 4066
15. Zhang, L., Cai, W. & Bao, N. Top-Level Design Strategy to Construct an Advanced High-Entropy Co–Cu–Fe–Mo (Oxy)Hydroxide Electrocatalyst for the Oxygen Evolution Reaction. *Adv. Mater.* 2021, **33**, 2100745
16. Duan, C. *et al.* Nanosized high entropy spinel oxide $(FeCoNiCrMn)_3O_4$ as a highly active and ultra-stable electrocatalyst for the oxygen evolution reaction. *Sustainable Energy Fuels* 2022, **6**, 1479
17. Zhang, Y. *et al.* Stabilizing Oxygen Vacancy in Entropy-Engineered $CoFe_2O_4$-Type Catalysts for Co-prosperity of Efficiency and Stability in an Oxygen Evolution Reaction. *ACS Appl. Mater. Interfaces* 2020, **12**, 29, 32548–32555

18. Zhang, Y., Dai, W., Zhang, P., Lu, T. & Pan, Y. In-situ electrochemical tuning of $(CoNiMnZnFe)_3O_{3.2}$ high-entropy oxide for efficient oxygen evolution reactions. *J. Alloys Compd.* 2021, **868**, 159064

19. Nguyen, T. X., Liao, Y. C., Lin, C. C., Su, Y. H. & Ting, J. M. Advanced High Entropy Perovskite Oxide Electrocatalyst for Oxygen Evolution Reaction. *Adv. Funct. Mater.* **31**, 2101632 (2021)

20. Sun, Y. *et al.* Covalency competition dominates the water oxidation structure–activity relationship on spinel oxides. *Nat. Catal.* 2020, **3**, 554-563

21. Shi, H. & Zhao, G. Water Oxidation on Spinel $NiCo_2O_4$ Nanoneedles Anode: Microstructures, Specific Surface Character, and the Enhanced Electrocatalytic Performance. *J. Phys. Chem. C* 2014, **118**, 45, 25939–25946

22. Landon, J. *et al.* Spectroscopic characterization of mixed Fe-Ni oxide electrocatalysts for the oxygen evolution reaction in alkaline electrolytes. *ACS Catal.* 2012, **2**, 8, 1793–1801

23. Han, S. *et al.* One-Step Electrodeposition of Nanocrystalline $Zn_xCo_{3-x}O_4$ Films with High Activity and Stability for Electrocatalytic Oxygen Evolution. *ACS Appl. Mater. Interfaces* 2017, **9**, 20, 17186–17194

24. Jiang, J., Zhang, A., Li, L. & Ai, L. Nickel-cobalt layered double hydroxide nanosheets as high-performance electrocatalyst for oxygen evolution reaction. *J. Power Sources* **278**, 445-451 (2015)

25. Zhang, Y., Cui, B., Zhao, C., Lin, H. & Li, J. Co-Ni layered double hydroxides for water oxidation in neutral electrolyte. *Phys. Chem. Chem. Phys.* 2013, **15**, 7363

26. Dong, C. *et al.* Rational design of cobalt-chromium layered double hydroxide as a highly efficient electrocatalyst for water oxidation. *J. Mater. Chem. A* 2016, **4**, 11292

27. Zou, X., Goswami, A. & Asefa, T. Efficient noble metal-free (electro)catalysis of water and alcohol oxidations by zinc-cobalt layered double hydroxide. *J. Am. Chem. Soc.* 2013, **135**, 46, 17242–17245

28. Yang, F. *et al.* Synergistic Effect of Cobalt and Iron in Layered Double Hydroxide Catalysts for the Oxygen Evolution Reaction. *ChemSusChem* **10**, 156-165 (2017)

29. Song, F. & Hu, X. Ultrathin cobalt-manganese layered double hydroxide is an efficient oxygen evolution catalyst. *J. Am. Chem. Soc.* 2014, **136**, 47, 16481-16484

30. Liang, Q., Brocks, G. & Bieberle-Hütter, A. Oxygen evolution reaction (OER) mechanism under alkaline and acidic conditions. *J. Phys. Energy* **3** (2021) 026001
